# Heavy metal exposure and ecological risk assessment in the Guizhou snub-nosed monkey (*Rhinopithecus brelichi*): a fecal biomonitoring study

**DOI:** 10.3389/fvets.2026.1897748

**Published:** 2026-08-12

**Authors:** Tianyou Yang, Haibo Li, Xiaodong Zhang

**Affiliations:** 1 College of A&F Engineering and Planning, Tongren University, Tongren, Guizhou, China; 2 Fanjingshan National Nature Reserve Administration, Tongren, Guizhou, China; 3 Guizhou Fanjingshan Forest Ecosystem National Observation and Research Station, Jiangkou, Guizhou, China

**Keywords:** bioaccumulation, ecological risk assessment, feces, Guizhou snub-nosed monkey, heavy metals

## Abstract

Within the “One Health” framework, terrestrial wildlife serves as an effective indicator species for monitoring various pollutants, including heavy metals. In this study, we aimed to characterize heavy metal exposure in Guizhou snub-nosed monkeys (*Rhinopithecus brelichi*) in the Fanjing Mountain World Natural Heritage Site (FMWNHS) in Guizhou Province, China. To this end, feces samples from Guizhou snub-nosed monkeys were collected at different points within the study area and the concentrations of six heavy metals (Cd, Cr, Cu, Pb, As, and Hg) were quantified. Specifically, concentrations of Cd, Cr, Cu, Pb, and As were determined using inductively coupled plasma mass spectrometry, whereas Hg was quantified by atomic fluorescence spectrometry. The bioaccumulation characteristics, ecological risks and health risk of these heavy metals were also determined based on their bioaccumulation factors, potential ecological risk indices and hazard quotient, respectively. Spearman correlation analysis, combined with non-negative matrix factorization and positive matrix factorization, was performed to determine the sources of these heavy metals and their contributions to the observed pollution levels. The results showed significant variations in the accumulation levels of the investigated heavy metals, with Cu showing the highest accumulation and distinct enrichment characteristics, whereas As and Hg showed the lowest accumulation levels. Ecological risk indices indicated moderate to very high ecological risks, with Niujiaodong and Liujiawan identified as high-risk areas. Hg and Cd were the primary contributors to the ecological risks. However, the hazard quotient values for all study areas were below 1, indicating that exposure to these heavy metals remained within the acceptable safety threshold and posed a low potential risk of adverse health effects in Guizhou snub-nosed monkeys. Source apportionment showed that Cd, As, Pb, and Cr primarily originated from composite pollution sources, Hg from atmospheric pollution sources, and Cu from natural geogenic sources. Overall, fecal based bioaccumulation analysis provides a simple, safe, and non-invasive approach for monitoring exposure to environmental pollutants and their effects on critically endangered species. The findings of this study may also be of great significance in conservation efforts for rare and endangered species.

## 1 Introduction

Presently, environmental pollution constitutes a critical global challenge and is widely recognized as a primary driver of biodiversity loss (1). Chemical pollutants damage the health of individuals, disrupt population stability, and increase the risk of extinction (2). Notably, heavy metals, characterized by high atomic weights or densities, are harmful to living organisms. Although some heavy metals are essential trace elements involved in various physiological processes, they can exert toxic effects at elevated concentrations. In contrast, non-essential heavy metals are toxic even at very low concentrations (3). Their persistence and continuous input from industrial emissions, agricultural runoff, and urban waste have made heavy metals widespread environmental pollutants (4). For animals, heavy metal exposure pathways, including respiration, foraging, rumination, and predation, lead to bioaccumulation in tissues and limited excretion. These heavy metals are persistent in the environment (5) and can adversely affect the health of wildlife individuals by inducing oxidative stress, disrupting reproduction, and altering behavior. Such effects may ultimately reduce individual fitness and population viability while increasing the risk of extinction (6, 7). Heavy metal pollution, which has become a critical issue in global wildlife conservation, is also strongly associated with animal, human, and environmental health, a concept encapsulated in the “One Health” framework (8).

The Guizhou snub-nosed monkey (*Rhinopithecus brelichi*), which belongs to genus *Rhinopithecus*, subfamily Colobinae, family Cercopithecidae, and order Primates (9), is primarily distributed within the Fanjing Mountains World Natural Heritage Site (FMWNHS) in northeastern Guizhou, China, and is an endemic flagship species of the region. The large elevation range in the FMWNHS has created a complex alpine gorge landscape, distinct from the surrounding low-elevation hills and low mountains and resembling an “island” rising from the sea. This “sky island” isolation has further restricted gene flow, and the population is threatened by habitat fragmentation, long reproductive cycles, and low genetic diversity (10). The species is listed as Critically Endangered (CR) on the IUCN Red List and is considered one of the world's most endangered primates.

To date, research on Guizhou snub-nosed monkeys has primarily focused on gut microbiota composition (11), dietary habits (9), population viability, and genetic structure (12), while their exposure to heavy metals remains unclear. To bridge this knowledge gap, the use of non-invasive methods is critical (13). Reportedly, feces are a suitable non-invasive specimen for investigating heavy metal exposure in wildlife (14). Typically, feces contain metals at high concentrations, and its excretion represents one of the major pathways for eliminating most or excessively consumed elements (15). Feces are also particularly important because its heavy metal content reflects heavy metal exposure via dietary intake (3). Additionally, feces analysis is effective for monitoring heavy metal exposure and the associated impacts on endangered species (7, 16). Although fecal monitoring has been widely applied in wildlife research, field studies of heavy metal contamination in non-human primates remain relatively limited. Therefore, in this study, this non-invasive sampling method was adopted to analyze heavy metal exposure in Guizhou snub-nosed monkeys in the FMWNHS. Our specific objectives were to: (1) determine the bioaccumulation characteristics of heavy metals in the feces of Guizhou snub-nosed monkeys; (2) clarify the status of heavy metal pollution in this species and assess associated ecological risks; and (3) conduct source apportionment for the identified fecal heavy metals. The findings of this study may provide valuable insights regarding heavy metal exposure in this species and support conservation efforts for this species as well as other endangered species.

## 2 Materials and methods

### 2.1 Study area

**Figure 1:**
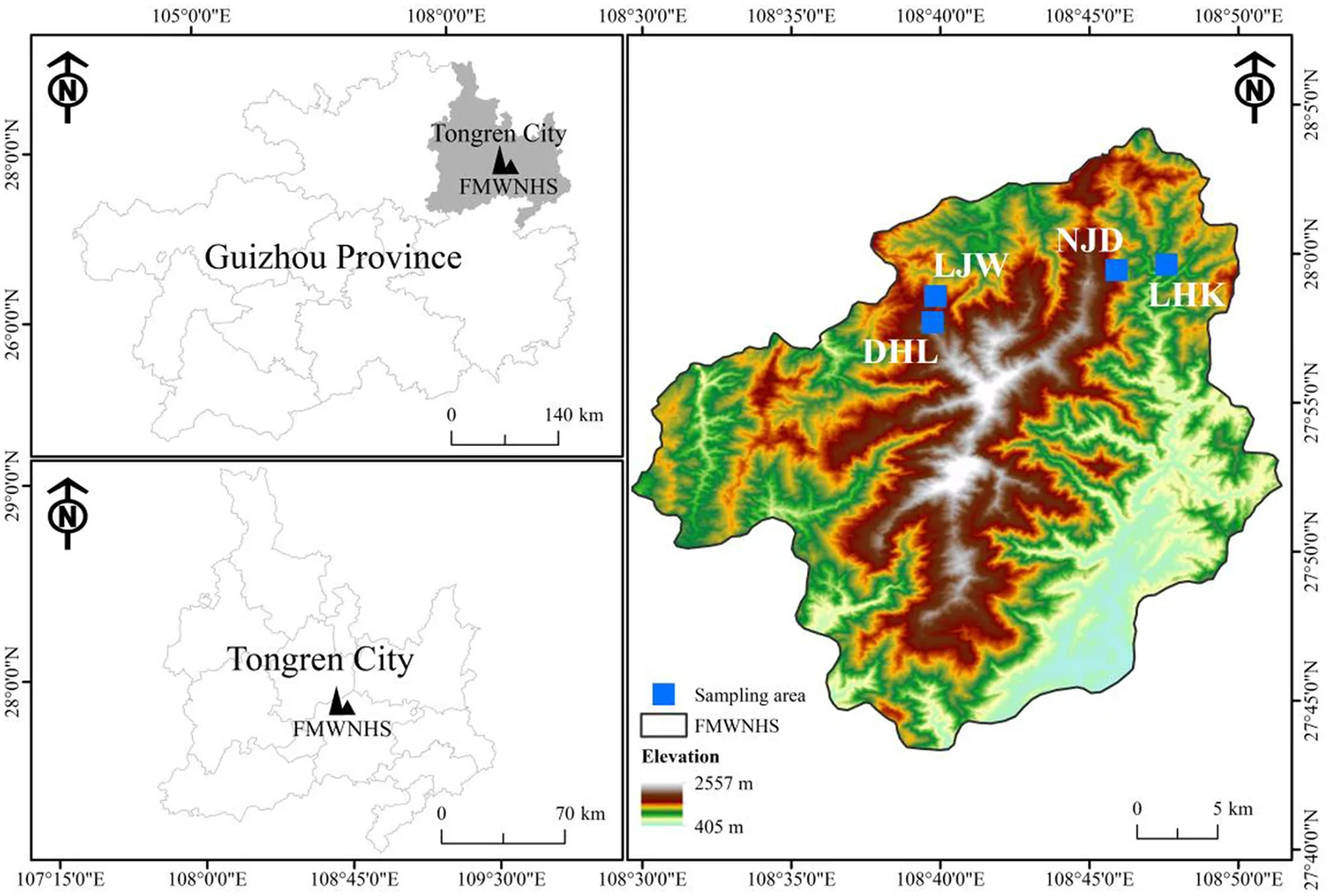
Map of study area showing sampling sites.

The FMWNHS (27°49′50″-28°01′30″N, 108°45′55″-108°48′30″E) is located at the junction of Jiangkou County, Yinjiang Tujia and Miao Autonomous County, and Songtao Miao Autonomous County in Tongren City, northeastern Guizhou Province, China (Figure 1). It covers an area of 775.14 km^2^ and is characterized by an East Asian monsoon climate, with mean annual temperature ranging between 6 °C and 17 °C. Further, in January and July, mean daily temperatures in this region vary in the ranges 3.1–5.1 and 15 °C−27 °C, respectively, and the accumulated active temperature ≥10 °C ranges from 1,500 °C to 5,500 °C. Mean annual precipitation in this region fluctuates between 1,100 and 2,600 mm, whereas humidity remains consistently high (above 80%) throughout the year (17). Owing to the rich and diverse vegetation of this mountainous area, it is considered as one of the most biodiverse regions in China. It is also the site with the best-preserved distinct primeval forest at this latitude (18), and in 2018, it was added to the UNESCO World Heritage List (19).

### 2.2 Sample collection and preparation

This study was conducted with permission from the Administrative Bureau of the FMWNHS, Guizhou Province, China and adhered strictly to the Laws of the People's Republic of China on the Protection of Wildlife. Samples were collected using non-invasive methods. Thus, there was no direct contact with or disturbance to the Guizhou snub-nosed monkeys.

Based on the known distribution pattern of Guizhou snub-nosed monkeys within the FMWNHS, fecal samples were collected between January and April 2025 from four distinct sites: Liujiawan (LJW), Niujiaodong (NJD), Dahuanlu (DHL), and Lianghekou (LHK). Ten fresh fecal samples were collected at each site. Field observations and video surveillance confirmed that the monkey groups inhabiting the four sampling sites were distinct social groups, each exhibiting relatively independent home ranges and movement patterns. A single-follow fecal sampling strategy was employed, in which fresh fecal samples were collected immediately after defecation by continuously tracking monkey groups. Monkey groups were located primarily through visual observation and monitored from a distance to minimize human disturbance. After a group left the area, researchers immediately entered to search for fecal samples. Only freshly deposited feces were collected, with freshness assessed based on fecal size and coloration. To minimize the likelihood of repeatedly sampling the same individual, samples were collected at intervals of more than 20 m, and only one fecal sample was obtained from each sampling location. Adjacent fecal deposits were not collected. To minimize contamination from environmental matrices, the surfaces of the collected samples were rinsed with deionized water and 70% ethanol to remove residual soil and plant debris. All the samples were subsequently stored in sealed polyethylene bags and frozen at −20 °C until laboratory analysis.

### 2.3 Laboratory analysis

At the laboratory, the collected fecal samples were oven-dried at 60 °C for 24 h, mechanically ground, and sieved using a 1-mm sieve. Heavy metal concentrations were measured by Hunan Huahuan Testing Technology Co., Ltd., China. Approximately 0.2–0.5 g of each sample (accurate to 0.0001 g) was weighed using an analytical balance and transferred into a polytetrafluoroethylene (PTFE) digestion vessel. Then, 5–10 mL of nitric acid was added, and the sealed vessel was allowed to stand for 12 h. The samples were digested in a microwave digestion system according to the standard protocol. After digestion, the solution was cooled, and the vessel was carefully vented by slowly opening the lid. The inner surface of the vessel lid was rinsed with a small volume of deionised water, and the digestion vessel was then placed on a temperature-controlled hot plate at 80 °C for 3–6 min to remove residual brown fumes. Finally, the digest was diluted to a final volume of 25 mL with deionised water. Concentrations of Cd, Cr, Cu, Pb, and As were measured using inductively coupled plasma mass spectrometry (ICP-MS; SUPEC 7000, Focused Photonics, China). Analytical procedures followed the Chinese National Food Safety Standard, Determination of Multi-Elements in Foods (GB 5009.268-2016). Hg was quantified by atomic fluorescence spectrometry (AFS; AFS-8520, Haiguang Instruments, Beijing, China), following the Chinese National Food Safety Standard, Determination of Total Mercury and Organic Mercury in Foods (GB 5009.17-2021).

All reagents were of analytical grade. A certified biological reference material (GBW10010, Beijing Tanmo Quality Inspection Technology Co., Ltd., China) was used for quality assurance/quality control (QA/QC) throughout the analytical procedure. Spike recovery rates ranged from 93 to 106%, and the relative standard deviation (RSD) was maintained below 5%. Analytical blanks and digestion blanks were included to verify accuracy and minimize potential contamination from laboratory materials and procedures. The limits of detection (LoD) for the analyzed heavy metals were 0.002 mg/kg for Cd and As, 0.05 mg/kg for Cr and Cu, 0.02 mg/kg for Pb, and 0.001 mg/kg for Hg.

### 2.4 Determination of bioaccumulation factors (BAFs)

The bioaccumulation factor (BAF) of a metal (metalloid) is defined as the ratio of the concentration of the metal (metalloid) in an organism or a specific tissues to that in the surrounding medium (20). This metric is often used to evaluate the relationship between the accumulation of a metal within an organism and its bioavailability in the environment (21). Guizhou snub-nosed monkey feeds predominantly on plant material, and the elemental composition of its feces primarily reflects dietary intake through ingested vegetation. The chemical elements present in herbivore feces can serve as indicators of dietary exposure. In this study, the ratios of heavy metal concentrations in feces to those in soil were calculated to characterize the monkeys' environmental exposure to heavy metals. In this study, it was calculated according to Equation 1.


$$\begin{array}{l} BAF=C_{f}/C_{s} \end{array}$$ (1)

where *C*_*f*_ represents the concentration of the heavy metal in the collected feces sample (mg/kg) and *C*_*s*_ represents the background concentration of the heavy metal in the soil (mg/kg). The *C*_*s*_ values were obtained from established research findings for the FMWNHS (22), and BAFs > 1 indicated a potential risk of bioaccumulation. Further, as previously determined, the soil contents of Cd, Cr, Cu, Pb, As, and Hg in the study area are 1.190, 64.700, 14.700, 37.700, 8.330, and 0.155 mg/kg, respectively (22).

### 2.5 Ecological risk assessment

The potential ecological risk index (*RI*) (23) was employed to evaluate the extent of heavy metal contamination and the associated potential ecological risks to Guizhou snub-nosed monkeys. This metric integrates multiple factors, including the physicochemical properties of heavy metals, their toxicological effects, and their synergistic interactions with other elements. In this study, it was calculated according to Equation 2 as follows (24):


$$\begin{array}{l} RI=\overset{n}{\underset{i=1}{\sum }}E_{r}^{i}=\overset{n}{\underset{i=1}{\sum }}T_{r}^{i}\times \frac{C^{i}}{C_{0}} \end{array}$$ (2)

where $E_{r}^{i}$ represents the potential ecological risk coefficient of heavy metal *i*, and reflects both the contamination level and potential ecological risk of the heavy metal, $T_{r}^{i}$ represents the toxic response factor of heavy metal *i* (30, 2, 5, 5, 10, and 40 for Cd, Cr, Cu, Pb, As, and Hg, respectively) (25), *C*^*i*^ denotes the concentration of heavy metal *i* in the fecal sample, and *C*_0_ denotes the background concentration of heavy metal *i* in the study area. The *C*_0_ values used in this study represent the average concentrations of the heavy metal based on 3 out of 40 fecal samples that showed the lowest heavy metal contents (26).

The grading of the $E_{r}^{i}$ values into five risk levels was as follows: $E_{r}^{i}$ < 40, low risk; 40 ≤ $E_{r}^{i}$ < 80, moderate risk; 80 ≤ $E_{r}^{i}$ < 160, considerable risk; 160 ≤ $E_{r}^{i}$ < 320, very high risk; and $E_{r}^{i}$ ≥ 320, extremely high risk. Further, the grading of the *RI* values into four levels was as follows: *RI* < 150, low risk; 150 ≤ *RI* < 300, moderate risk; 300 ≤ *RI* < 600, considerable risk; and *RI* ≥ 600, very high risk (25).

### 2.6 Health risk assessment

The hazard quotient (*HQ*) was used to evaluate non-carcinogenic risk from heavy metals, following USEPA guidelines as used in previous studies on captive primates (7) and golden takins (*Budorcas taxicolor bedfordi*) (27). The *HQ* was calculated according to Equation 3.


$$\begin{array}{l} HQ=\frac{ADD_{dod}}{RfD} \end{array}$$ (3)

where ADD_dod_ denotes the average daily oral dose (ng/g·d), and RfD denotes the human reference dose. The RfD values for Cd, Cr, Cu, Pb, As, and Hg, as established by the U.S. Environmental Protection Agency (28), are 1, 1500, 40, 3.5, 0.06, and 0.3 μg kg^−1^ bw day^−1^, respectively. An *HQ* below 1 indicates that exposure remains within safe limits. An *HQ* between 1 and 10 suggests potential adverse health effects and warrants further evaluation. Higher *HQ* values indicate proportionally greater health risk.

The average daily oral dose (ADD_dod_) was calculated using the Equation 4.


$$\begin{array}{l} ADD_{dod}=\frac{C_{F}\times F_{d}\times T_{d}}{\left[\left(1-P_{a}\right)+\left(P_{a}\times P_{e}\right)\right]\times BW} \end{array}$$ (4)

where C_F_ denotes the fecal heavy metal concentration. F_d_ is the mean single-defecation mass (g/time, dry weight), and T_d_ is the defecation frequency (times/day). Because direct field measurement of F_d_ and T_d_ was not feasible and no published values are available for Guizhou snub-nosed monkeys, we used data from the congeneric golden snub-nosed monkeys (*Rhinopithecus roxellana*) (F_d_ = 18.5 g/time; T_d_ = 7 times/day). P_a_ denotes the gastrointestinal absorption rate (%) of each element in animals or humans, and P_e_ denotes the proportion (%) of ingested element excreted in feces. Following the International Programme on Chemical Safety (29), P_a_ values for Cd, Cr, Cu, Pb, As, and Hg were set at 5%, 5%, 50%, 10%, 30%, and 7%, respectively. P_e_ values for Cr, Cu, Pb, As, and Hg were set at 2%, 80%, 16%, 8%, and 60%, respectively; for Cd, P_e_ was set at 90% based on a previous human fecal study (30). BW denotes body weight (kg). Because age and sex were not determined in this study, the average body weight of Guizhou snub-nosed monkeys was assumed to be 11.5 kg (31).

### 2.7 Heavy metal source apportionment

To identify the sources of the investigated heavy metals and contributions, source apportionment analysis was realized via non-negative matrix factorization (NMF) using the NMF package (version 0.26) in R version 4.5.1 (R Core Team, Vienna, Austria). The optimal number of factors in the model was determined to be three.

We used positive matrix factorization (PMF) as a complementary source-apportionment method. PMF is a receptor-based technique that identifies pollution sources qualitatively and estimates their contributions quantitatively (32). We performed all PMF modeling with the U.S. Environmental Protection Agency PMF model (EPA PMF 5.0). The signal-to-noise (S/N) ratios of all heavy metals exceeded 8 and were therefore classified as “strong” variables.

PMF models with two to five factors were evaluated, and each factor solution was run 20 times using different random seeds. The optimal number of factors was determined by jointly considering the trend in Q values, the chemical interpretability of the factor profiles, and the statistical stability of the solutions. The Q(Robust) values decreased with increasing factor number as follows: 1,296.6 for the two-factor solution, 721.9 for the three-factor solution (48% reduction), 333.2 for the four-factor solution (55% reduction), and 109.9 for the five-factor solution (70% reduction). Because only six heavy metals were included in the analysis, no distinct inflection point was observed in the Q values. Therefore, the final selection of the number of factors was based primarily on the chemical interpretability of the factor profiles, with the magnitude of the *Q*-value reduction used as a supplementary criterion. The five-factor solution produced a “full-spectrum” factor, in which all six elements exhibited loadings ranging from 20 to 80%, indicating clear overfitting (element-to-factor ratio = 1.2). In the four-factor solution, Pb was distributed across all four factors, with a maximum loading of only 37% on any single factor, indicating no distinct source attribution. In contrast, the three-factor solution yielded well-defined dominant elemental assemblages for each factor, and the resulting factor profiles exhibited clear geochemical significance. Accordingly, the three-factor solution was selected for the final source apportionment analysis.

Model robustness was evaluated using four complementary diagnostic procedures applied to the converged base run. Bootstrap analysis (100 runs; minimum correlation coefficient, *R* = 0.6) showed mapping rates of 100% for Factor 2 and 97% for Factor 3, both exceeding the recommended threshold of 80%. Factor 1 achieved a mapping rate of 65%, indicating greater sensitivity to high-leverage samples. The base-run Q(Robust) value of 721.9 fell within the interquartile range (25^th^-75th percentile: 407–1108) of the Bootstrap distribution, supporting the overall stability of the solution. DISP analysis identified no factor swaps at any of the four dQmax levels (4, 8, 15, and 25), with an Error Code of 0 and a maximum Q displacement of −0.081, indicating the absence of rotational ambiguity. Fpeak rotation analysis further demonstrated that all tested Fpeak values (−1.0 to +1.5) increased Q(Robust) by 11.2%−78.5% relative to the base solution, confirming that Fpeak = 0 provided the optimal solution and that no oblique rotation was required. Model performance was further assessed by comparing observed and PMF-predicted concentrations. The coefficients of determination (*R*^2^) were 0.955 for Hg, 0.776 for Pb, 0.747 for Cr, and 0.747 for Cd, all exceeding the threshold of 0.70 commonly adopted in PMF studies. In contrast, the *R*^2^ values for As and Cu were 0.510 and 0.418, respectively, indicating that the model had relatively limited explanatory power for these two elements. Standardized residuals were examined for each sample and element. All standardized residuals for Hg fell within the ±3 acceptance range, with a maximum value of 2.13, and the corresponding Q(true)/Q(exp) ratio was 1.04, indicating excellent model fit. In contrast, Cd, Pb, and As each exhibited a small number of samples with standardized residuals exceeding the ±3 criterion, with Q(true)/Q(exp) ratios of 13.86, 14.00, and 10.50, respectively. The corresponding Q(true)/Q(exp) ratios were 4.57 for Cu and 6.74 for Cr.

### 2.8 Statistical analysis

All statistical analyses were performed using SPSS software v27.0 (IBM, Armonk, NY, USA), and for figure generation, Origin 2024 software (OriginLab, Northampton, MA, USA) was employed. Owing to the non-normal distribution of the data (Shapiro-Wilk test, *P* < 0.05), the non-parametric Kruskal-Wallis test was employed to determine differences in heavy metal concentrations. Spearman's rank correlation analysis was also performed to examine the relationships among the heavy metal elements.

## 3 Results

### 3.1 Characteristics of heavy metals in feces samples

**Figure 2:**
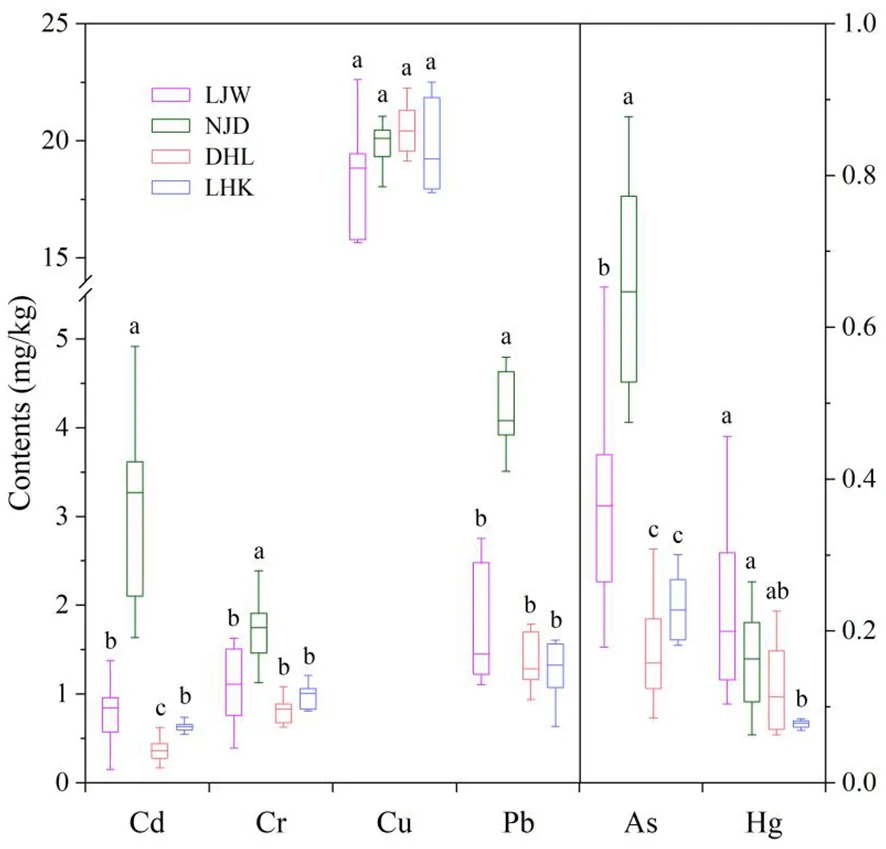
Heavy metal concentrations in feces samples. Boxplots show the median (horizontal line), interquartile range (box edges), and whiskers (1.5 × IQR). The different lowercase letters indicate significant regional differences in mean heavy metal contents (*P* < 0.05).

The accumulation levels of the target heavy metals in the collected feces samples varied considerably as shown in Figure 2. Cu exhibited the highest relative concentration across sites, whereas As and Hg showed the lowest concentrations. The concentration trend was: Cu > Pb > Cr > Cd > As > Hg, and notably, this trend was consistent across three sampling sites (LJW, DHL, and LHK), and only changed slightly at NJD (Cu > Pb > Cd > Cr > As > Hg).

The heavy metals also showed distinct spatial patterns. Except for Cu (*H* = 3.443, df = 3, *P* = 0.328), the other heavy metals showed significant differences in terms of concentrations across the sampling locations (*P* < 0.05). The concentrations of Pb (H = 22.891, df = 3, *P* < 0.001), Cd (H = 27.970, df = 3, *P* < 0.001), Cr (H = 19.594, df = 3, *P* < 0.001), and As (H = 25.033, df = 3, *P* < 0.001) in NJD samples were significantly higher than those in LJW, DHL, and LHK samples. Additionally, Hg (H = 16.437, df = 3, *P* < 0.001) concentrations in LJW and NJD samples were significantly higher than those in LHK samples.

### 3.2 Heavy metal bioaccumulation risk

**Figure 3:**
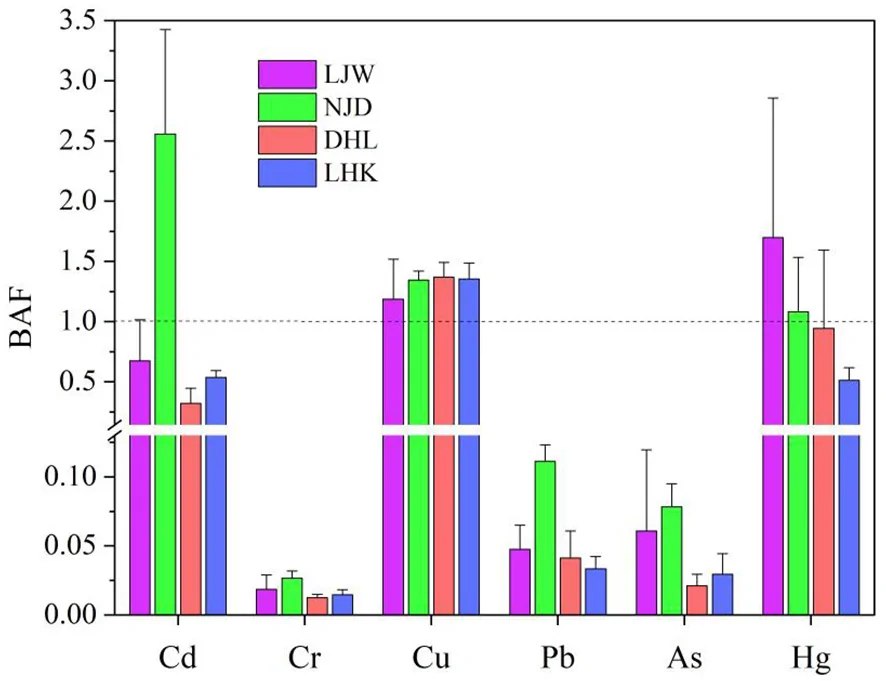
Bioaccumulation factors of heavy metals in feces relative their values in soil.

To evaluate environmental exposure to heavy metals, the BAFs for individual heavy metals were calculated as the ratios of their concentrations in feces to those in soil (Figure 3). Notably, the heavy metals showed different bioaccumulation characteristics. Cr, As, and Pb showed relatively low bioaccumulation levels, with mean BAFs < 1 across all locations. Conversely, Cu exhibited potential bioaccumulation risk, showing with BAFs > 1 across all sampling sites. Additionally, the bioaccumulation risk of Cd was the highest at NJD (BAF = 2.56), whereas Hg posed a bioaccumulation risk at two sites, LJW and NJD.

### 3.3 Ecological risk assessment

**Figure 4:**
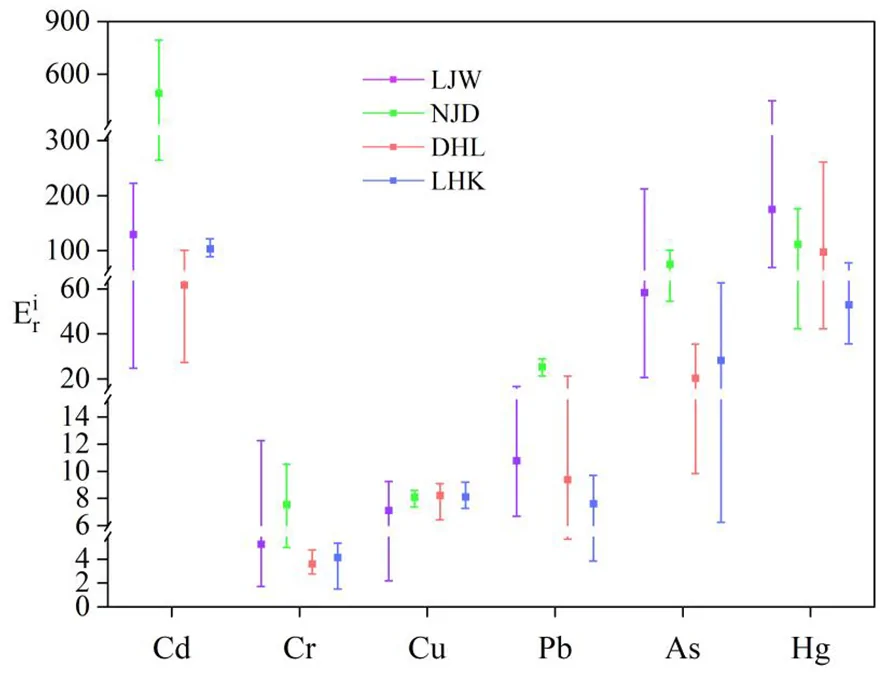
Potential ecological risk coefficients ($E_{r}^{i}$) of heavy metals in feces. Squares represent the mean values, and whiskers indicate the data range (minimum-maximum).

**Figure 5:**
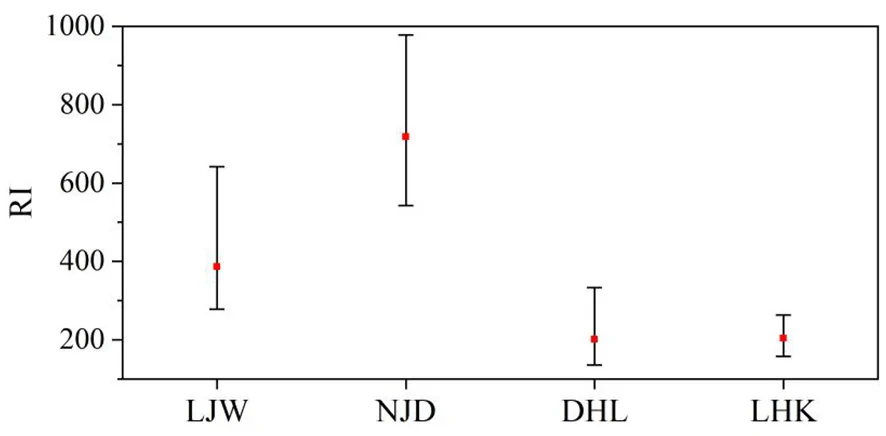
Potential ecological risk index (*RI*) values of heavy metals in feces samples. Squares represent the mean values, and whiskers indicate the data range (minimum-maximum).

The ecological risks of the heavy metals were assessed in terms of their $E_{r}^{i}$ (Figure 4) and *RI* (Figure 5) values. Based on the $E_{r}^{i}$ values, the pollution levels of the heavy metals varied considerably by sampling site. The mean $E_{r}^{i}$ values for Cr, Cu, and Pb were consistently < 40 across the different sites, implying low levels of pollution as well as low potential ecological risks. Further, as shown, the risk at DHL and LHK is moderate, and at LJW and NJD it is low. Conversely, Cd and Hg posed high potential ecological risks (moderate and above) across all the sampling sites, and specifically showed very high potential ecological risks at NJD and LJW, respectively.

To further comprehensively evaluate the potential ecological risks of the six heavy metals to Guizhou snub-nosed monkeys in different regions, *RI* values were calculated. Thus, we observed relatively high mean *RI* values for all the heavy metals across the four sampling regions. The *RI* values showed moderate risk at DHL and LHK, high risk at LJW, and very high risks at NJD. These findings indicated that heavy metal pollution in the region may be severe, posing a high potential ecological risk to Guizhou snub-nosed monkeys.

In summary, although Cu exhibited the highest concentration, its $E_{r}^{i}$ was low, indicating a low level of potential ecological risk. The extent of heavy metal contamination and the associated comprehensive potential ecological risks to Guizhou snub-nosed monkeys were high. Serious Cd and Hg pollution were observed in certain regions, implying that these two heavy metals are the primary drivers of potential ecological risk to Guizhou snub-nosed monkeys in the study area.

### 3.4 Health risk assessment

**Figure 6:**
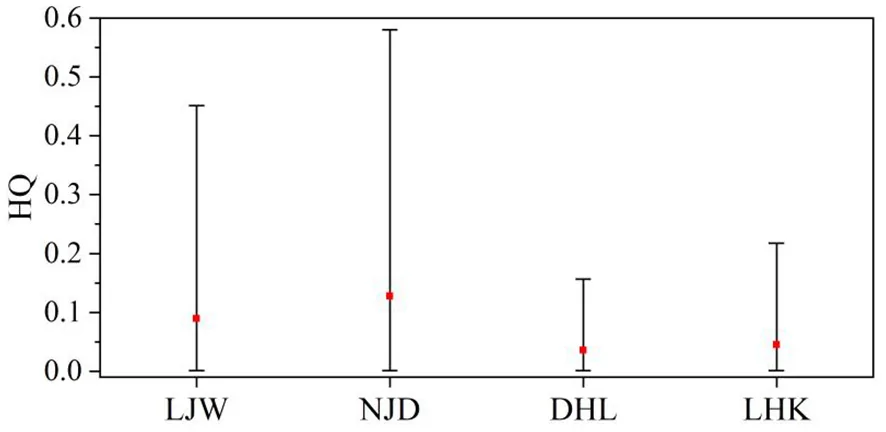
Estimated hazard quotient (HQ) values. Squares represent the mean values, and whiskers indicate the data range (minimum-maximum).

To assess regional health risks to Guizhou snub-nosed monkeys, we calculated HQ values for all six heavy metals in each of the four regions (Figure 6). All HQ values fell below 1, indicating that exposure to all heavy metals remained within safe limits.

### 3.5 Source analysis of heavy metals

**Figure 7:**
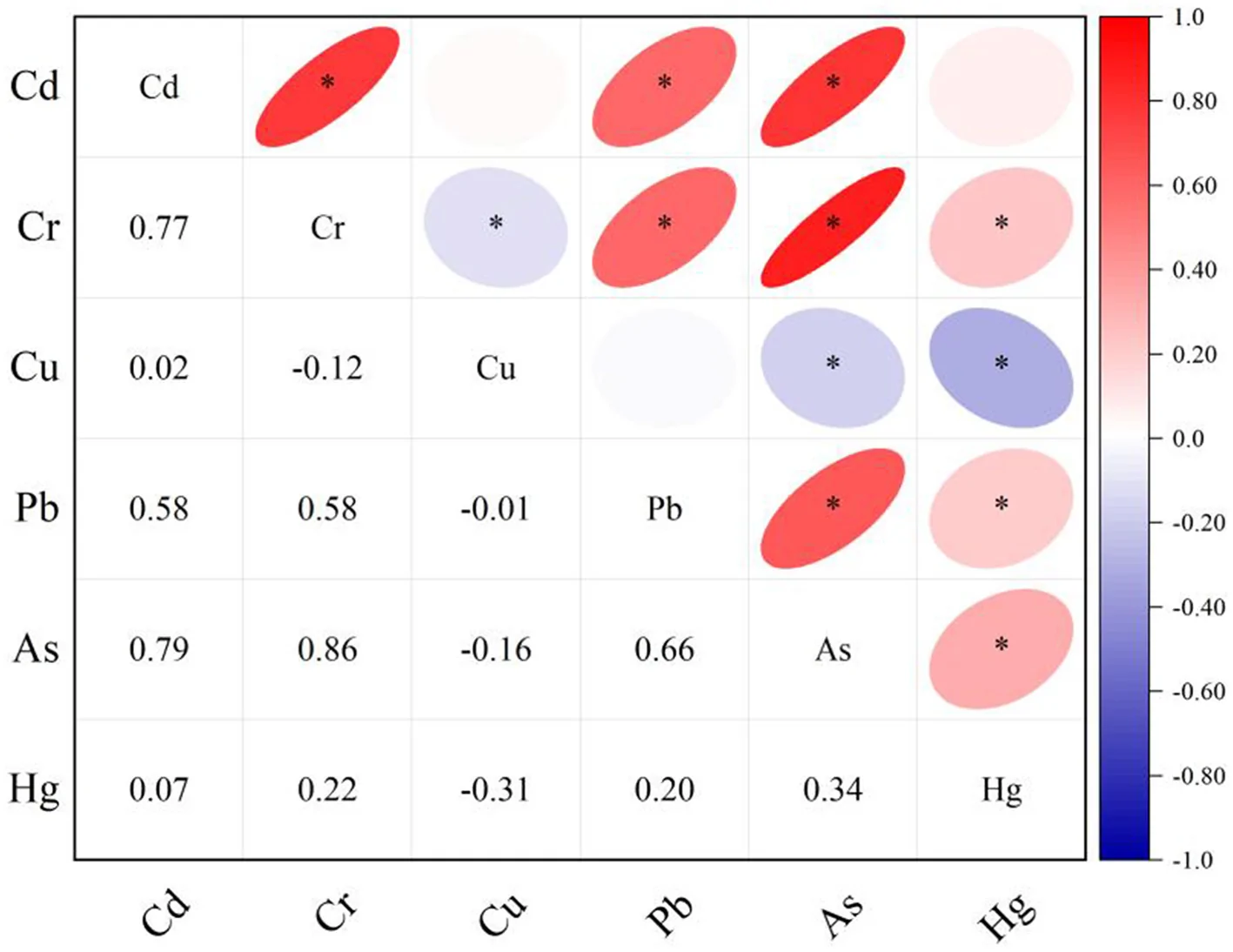
Correlations among heavy metals in feces. Correlation coefficients are presented in the lower triangle. The upper triangle displays the direction and strength of correlations using colored ellipses, with ellipse shape and color corresponding to the magnitude and sign of the correlation coefficients. Red indicates positive correlations, whereas blue indicates negative correlations. * denotes statistical significant differences at *P* ≤ 0.05.

Spearman correlation analysis was performed to capture potentially shared origins for the different heavy metals. The results obtained (Figure 7) showed similar origins for elements showing strong positive correlations. Notably, significant positive correlations (*P* < 0.05) were observed between Cd and Cr (0.77), Cd and Pb (0.58), Cd and As (0.79), Cr and Pb (0.58), Cr and As (0.86), Pb and As (0.66), and As and Hg (0.34), implying that these elements may have originated from shared or similar sources.

**Figure 8:**
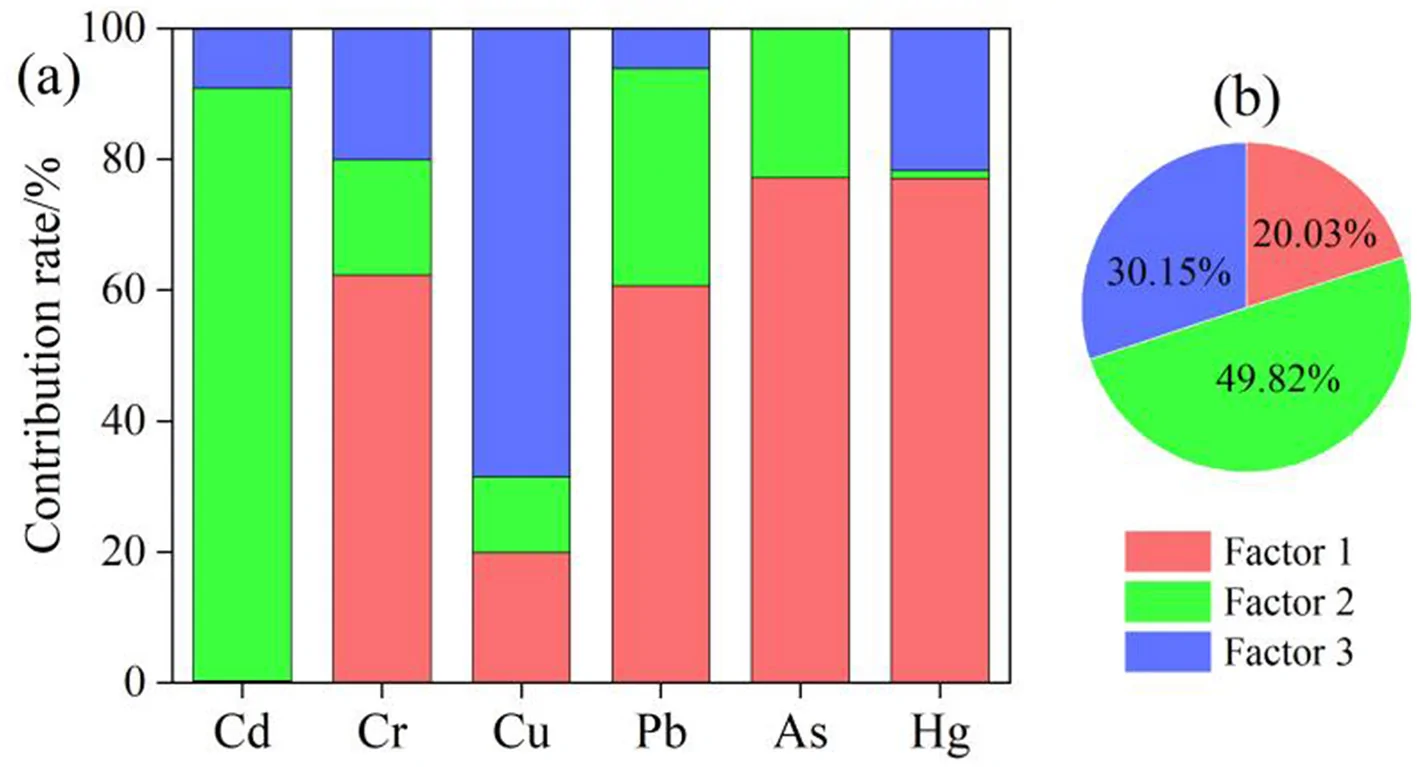
Source apportionment of heavy metals based on non-negative matrix factorization (NMF). **(a)** Relative contribution rate of each source to the heavy metals; **(b)** Average contribution rate of each source.

To further elucidate the interrelationships and environmental sources of these heavy metals, NMF was performed, and source apportionment was realized for each heavy metal (Figure 8). Thus, we observed that Factor 1 accounted for a contribution rate of 20.03% and was characterized by high As (77.16%), Hg (77.12%), Cr (62.32%), and Pb (60.64%) loadings. Factor 2 accounted for the highest contribution rate (49.82%), with Cd loading (90.60%) showing predominance. Additionally, Factor 3 accounted for a contribution rate of 30.15%, with Cu loading showing predominance (68.56%).

**Figure 9:**
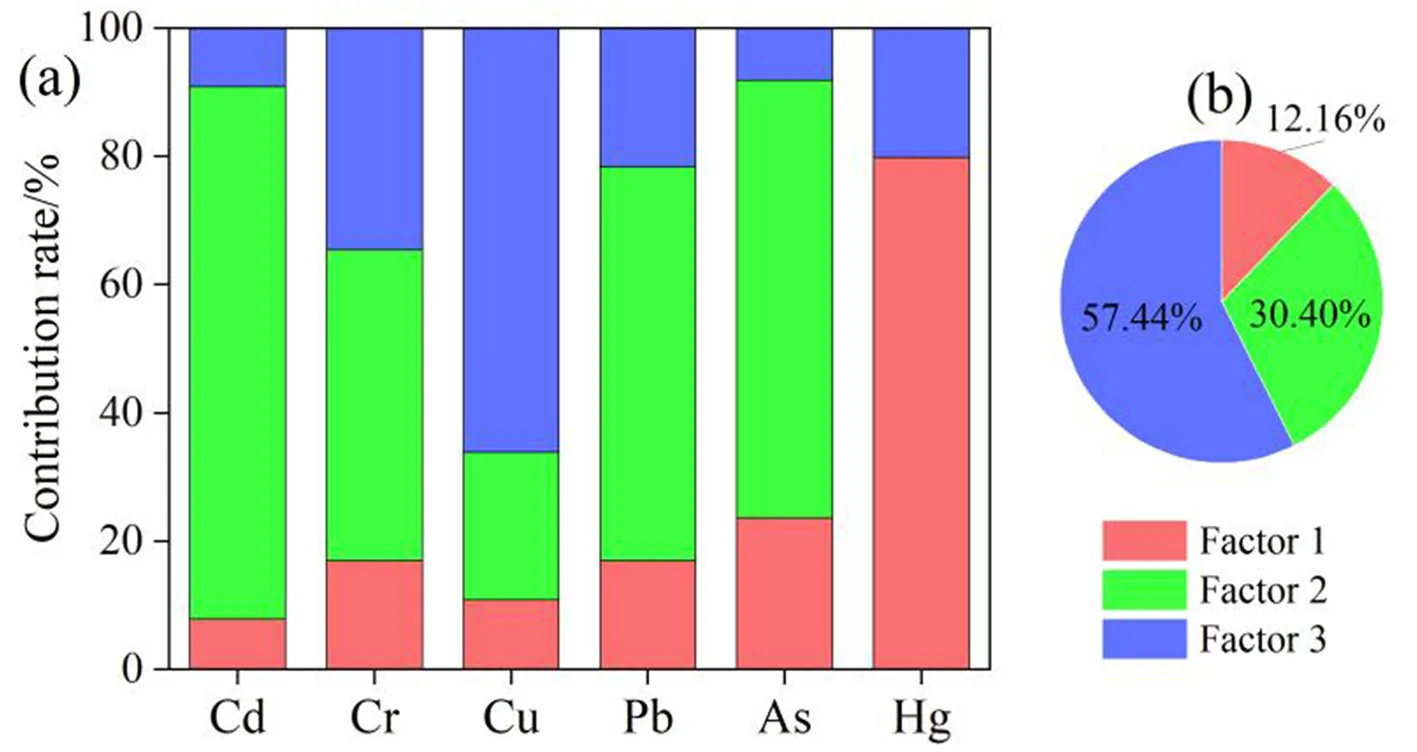
Source apportionment of heavy metals based on positive matrix factorization (PMF). **(a)** Relative contribution rate of each source to the heavy metals; **(b)** Average contribution rate of each source.

We performed source apportionment using PMF, which provided complementary information for identifying and quantifying environmental sources (Figure 9). Factor 1 accounted for 12.16% of the total contribution and was dominated by Hg (79.82%). Factor 2 contributed 30.40% and had high loadings of Cd (83.06%), As (68.23%), Pb (61.39%), and Cr (48.39%). Factor 3, accounting for 57.44% of the total contribution, was dominated by Cu (66.18%).

## 4 Discussion

Fecal analysis, as a non-invasive technique, minimizes injury and stress to wildlife, preserves ecosystem integrity, and adheres to ethical principles (8). Thus, it is suitable for studying trophic transfer as well as the bioaccumulation of hazardous substances in ecosystems (3). Relative to other non-invasive methods, such as saliva and hair analyses, fecal analysis is particularly advantageous for monitoring contamination profiles in endangered wildlife, which are generally difficult to monitor directly (33). Owing to their versatility and ease of collection, feces have become the most widely used specimen for the non-invasive monitoring of wildlife (34, 35). This is especially pertinent for the Guizhou snub-nosed monkey, a globally endangered primate characterized by a cryptic nature, as feces collection requires no direct contact with individuals and does not interfere with their behavioral activities (9). In this study, six heavy metals were detected in all the collected fecal samples, confirming the effectiveness of this non-invasive sampling approach.

### 4.1 Bioaccumulation characteristics of heavy metals in feces

Further, consistently high Cu concentrations were observed across all sampling sites, possibly owing to the predominantly herbivorous diet of Guizhou snub-nosed monkeys (9). To our knowledge, this is the first study to quantify heavy metal concentrations in the feces of Guizhou snub-nosed monkeys, so direct comparison is only possible with other species. The mean Cu concentration in Guizhou snub-nosed monkey feces (19.31 mg/kg) was lower than that reported for both wild and captive golden snub-nosed monkeys (36); although exact values were not reported, the figures in that study indicate Cu concentrations above 20.00 mg/kg. This concentration was also lower than that reported in golden takin feces (23.84 mg/kg) (27). These differences may reflect interspecific variation and differing environmental backgrounds. For herbivores, dietary intake is a direct source of trace metals (15) and the use of Cu as an essential micronutrient also supports its significantly higher concentration in the analyzed samples. The different sites showed no significant differences in fecal Cu levels (*P* > 0.05) possibly owing to their similar environmental background. This conclusion is consistent with previous reports that soil Cu content and distribution in the Fanjing Mountains do not vary significantly across altitudinal gradients (37). Conversely, the concentrations of the other heavy metals varied considerably across the different sampling sites possibly owing to habitat heterogeneity (38). Sampling sites NJD and LJW are located in the vicinity of historical mining areas, and this proximity possibly explains their relatively higher Pb, Cd, Cr, As, and Hg concentrations, as mining activities constitute a major source of heavy metal pollution (39).

Guizhou snub-nosed monkey feeds predominantly on plant materials, with leaves, buds, fruits, and flowers constituting its primary diet throughout the year (9). In herbivorous mammals, exposure to chemical elements occurs primarily through dietary intake. Consequently, the elemental composition of feces largely reflects ingested plant materials and consists mainly of the unassimilated fraction of recently consumed food, together with secretions from the anal glands (27). Pollutants typically biomagnify along food chains (40), and their BAFs represent the capacity of organisms to bioaccumulate them following environmental exposure (41). Because herbivore feces can serve as a biomonitor of heavy metal exposure, BAFs were used to assess heavy metal exposure in the Guizhou snub-nosed monkey. Given that our BAF estimates rely on historical soil concentration values (C_s_) from the literature, they should be regarded as a reference-based assessment with inherent limitations tied to the regional soil background. In this study, Cu exhibited distinct enrichment characteristics in Guizhou snub-nosed monkeys across the different sampling sites. Hg showed significant enrichment only at LJW and NJD, and Cd exhibited the highest BAF (2.56) at NJD, implying a certain degree of exposure risk associated with these elements.

### 4.2 Ecological risk assessment of heavy metals and sampling sites

However, these metals are different in terms of their toxic and synergistic effects, and their *RI* values can be used to capture their pollution levels and potential ecological risks, linking them to their toxicological consequences (23). The *RI* has been widely used to assess contamination risks in environmental media such as soil and air. In the present study, this index was adapted to evaluate the potential ecological risks associated with heavy metal concentrations in fecal samples. Because the heavy metals detected in Guizhou snub-nosed monkey feces are derived primarily from orally ingested vegetation, and no background concentrations were available for the corresponding plant materials, the mean concentration of the three samples with the lowest values for each element was used as the background value for RI calculation. This approach has been adopted in previous studies (26). In addition, using soil background values could introduce bias into the risk assessment because biological accumulation alters elemental concentrations in fecal samples relative to those in the surrounding environment. Therefore, given the distinct conceptual bases of the BAF and *RI*, different background reference systems were used for these two indices. Although the concentrations of Hg and Cd in the collected feces samples were relatively low, their pollution levels and potential ecological risks were relatively high. Conversely, Cu and Pb showed relatively low pollution and potential ecological risks while their concentrations in the samples were generally high. This discrepancy can be attributed to differences in their toxic response factors (24), which are particularly high for elements such as Hg and Cd. The mean *RI* values of the heavy metals for the Guizhou snub-nosed monkeys across the different regions all corresponded to moderate or high ecological risks, implying pollution and relative enrichment in the regional forest soils (42). Previous studies have indicated that the concentrations of elements with high toxic response factors influence their *RI* values (43). In this study, Hg and Cd were identified as the primary determinants of the observed high regional *RI* values, implying that they pose high ecological risks in the region. Relatively elevated concentrations of multiple heavy metals were also observed at the NJD and LJW, which may have contributed to the higher potential ecological risks identified in these two areas.

Accordingly, a long-term fecal-based heavy metal monitoring program should be established in the FMWNHS, with particular emphasis on monitoring Hg and Cd exposures to develop a continuous ecological risk database. The NJD and LJW should be designated as priority monitoring areas, with increased monitoring frequency for environmental media, including soils, water, and vegetation, as well as for wildlife, to strengthen the environmental risk early warning system. In addition, an integrated multi-compartment monitoring framework linking soils, vegetation, and Guizhou snub-nosed monkey should be developed to clarify the environmental transfer pathways of Hg and Cd, thereby providing a scientific basis for the conservation and management of endangered wildlife.

### 4.3 Health risk assessment of Guizhou snub-nosed monkey

However, the *HQ* values for all study areas were below 1, indicating that exposure to these heavy metals remained within the acceptable safety threshold and posed a low potential risk of adverse health effects in Guizhou snub-nosed monkeys. The HQ evaluates the potential adverse effects of heavy metals by comparing the estimated exposure dose with a reference safe dose and has been widely used to quantify health risks associated with dietary intake and environmental contaminant exposure, primarily in humans (38, 44). In the present study, this approach was adapted to assess the potential health risks of heavy metal exposure in the Guizhou snub-nosed monkey, a non-human primate. Because of the unique characteristics of the study species, all parameters used in the HQ calculations were derived from published studies on animals or humans, which may introduce some uncertainty into the assessment. The values for Fd and Td were based on a one-week observational study of captive golden snub-nosed monkeys (7), which provides a reasonable surrogate because the two species belong to the same genus. The P_a_ and P_e_ were adopted primarily from the standard values recommended by the International Programme on Chemical Safety, which are widely accepted for both human and animal risk assessments. The HQ approach has previously been applied in pollution studies using animal feces as the exposure matrix (7, 27). In captive adult primates, approximately 85% of sampled individuals had HQ values exceeding 1, whereas HQ values for As and Pb exceeded 1 in golden takins. When toxicological data for threatened or endangered species are unavailable, cross-species extrapolation using surrogate species is a common practice in ecological risk assessment (45), and the same approach was adopted in this study. Because the individual's age and sex could not be determined, an average body weight was used in the HQ calculations. Nevertheless, all estimated HQ values were below 1, suggesting that the HQ framework is applicable for assessing the potential health risks of heavy metal exposure in the Guizhou snub-nosed monkey.

### 4.4 Source analysis of heavy metals

Guizhou Province is rich in mineral resources and has a history of mining and smelting that spans thousands of years (46). Heavy metals released into the environment can be transported over long distances (47), and their circulation within isolated forest ecosystems in Karst regions, without major point sources of pollution, increases the risk of bioaccumulation (48). The NMF results suggested a common source for Hg, As, Cr, and Pb. However, Hg showed a significant positive correlation only with As (*P* < 0.05), and the PMF results further indicated that the source of Hg differed from those of the other elements. Therefore, based on the combined evidence from correlation analysis, NMF, and PMF source apportionment, Cd, As, Pb, and Cr were inferred to share a high degree of source similarity, whereas Hg likely originated from a different source. Mining activities, coal combustion, and ore distillation processes have also been shown to release As into the atmosphere (49). In this study, the NJD and LJW sampling sites were located near historical mining areas. Atmospheric deposition owing to mining, selection, and smelting activities associated with surrounding Hg, Mn, and Pb-Zn mines has also been suggested as the primary regional source of these elements (50). Regional studies have also demonstrated that Cr, Cd, Pb, Hg, and As in Fanjing Mountain Forest soils mainly originate from anthropogenic pollution via atmospheric deposition (42), consistent with the significant positive correlations observed between Cd and Cr, Pb, and As (*P* < 0.05) in this study. However, Cd pollution is strongly influenced by human activities, notably agriculture and industry. Therefore, the Cd, As, Pb, and Cr observed in this study may have originated from multiple combined pathways, implying that Factor 1 may be interpreted as “composite pollution sources.” Hg mining and coal combustion are widely recognized as the primary anthropogenic sources of environmental Hg. Owing to its high vapor pressure, Hg occurs predominantly as elemental Hg vapor in the atmosphere and can undergo long-range atmospheric transport (51). Previous studies have shown that global sources primarily influenced atmospheric Hg pollution in the Fanjing mountain region before the mid-20th century, whereas the contribution of regional sources has increased substantially since then (52). Hg in local soils has been attributed mainly to anthropogenic atmospheric deposition (42). The FMWNHS is located approximately 60 km northwest of the Wanshan Mercury Mine, the former-largest Hg production center in China, with a mining history spanning more than 600 years (53). This legacy mining area is therefore a plausible regional source of Hg contamination. Hg was likely derived primarily from atmospheric deposition, Factor 2 was interpreted as “atmospheric pollution sources.” Cu is derived primarily from natural geogenic sources and agricultural activities (54). Elevation has no significant effect on the distribution of Cu in Fanjing Mountain soil, as only minimal variations in Cu concentrations are observed across different soil layers (37). Soil Cu primarily originates from the weathering of parent materials rather than from surrounding industrial pollution (42). Consistent with these previous reports, no significant correlations were observed between Cu and the other heavy metals in this study. Both NMF and PMF source analysis also indicated a different origin for Cu. Considering the correlation between Cu in the feces samples and local geochemical background levels (55), Factor 3 was interpreted as “natural geogenic sources.”

### 4.5 Limitations

The fecal samples analyzed in this study were collected between January and April 2025 and therefore did not encompass a complete annual cycle. Heavy metal exposure in wildlife may vary seasonally (56). Although insects constitute a relatively larger proportion of the diet of Guizhou snub-nosed monkey during spring and winter (9), plant materials remain the dominant food resource throughout the year, with insects serving only as a supplementary dietary component despite their high energy and protein content (9). Consequently, the heavy metal concentrations reported here may not fully represent annual exposure patterns in this species. Multi-season sampling should be incorporated into future studies to characterize temporal variation in heavy metal exposure better.

Another limitation is the lack of species-specific toxicological and physiological parameters for the endangered Guizhou snub-nosed monkey. Owing to ethical and conservation constraints, direct experimental determination of parameters such as P_a_ and P_e_ is not feasible for this species. Consequently, studies assessing contaminant risks in threatened and endangered wildlife commonly rely on data from surrogate species (45). However, interspecific differences in physiology, metabolism, diet, and toxicokinetics may introduce uncertainty into cross-species extrapolation (57). The health risk estimates presented here should therefore be regarded as approximations rather than species-specific assessments. Because fecal samples were collected non-invasively, the age, sex, and identity of individual animals could not be determined, and an average body weight was used in the HQ calculations. Guizhou snub-nosed monkey exhibits pronounced sexual dimorphism, with adult males substantially heavier than females (31). As body weight is a key parameter in exposure assessment, using a single average value may introduce uncertainty into individual-level risk estimates. Nevertheless, because the objective of this study was to evaluate health risks at the population level rather than for individual animals, using an average body weight is a reasonable approximation given the available data. Future studies integrating non-invasive individual identification or demographic information would improve the accuracy of health risk assessments.

Field sampling was inherently challenging because the Guizhou snub-nosed monkey inhabits steep terrain with dense vegetation and frequent fog, which limited the sample size. Fecal samples were collected during single-tracking events to maximize spatial and temporal coverage across the study area. Although these procedures could not eliminate the possibility of resampling the same individual, they were designed to minimize pseudoreplication. Individual genetic identification was not performed due to financial constraints. Therefore, multiple samples from the same individual cannot be entirely excluded. Despite this limitation, the current sample size was sufficient to detect spatial variation for most heavy metals. Future studies incorporating non-invasive genetic identification would provide more robust assessments of heavy metal exposure and associated ecological risks.

Soil samples were not collected concurrently with fecal sampling. Instead, previously published soil data from the FMWNHS were used as the environmental reference for calculating the BAFs (22). That study systematically characterized regional soil heavy metal concentrations and spatial distributions by sampling multiple soil types at 100-m elevation intervals. Although these data provide an appropriate environmental baseline for evaluating heavy metal exposure through BAFs, they do not permit direct quantification of the relationship between fecal metal concentrations and local environmental contamination. Concurrent collection of environmental media in future investigations would strengthen the linkage between wildlife exposure and site-specific pollution sources.

The relatively small sample size may also have increased uncertainty in source apportionment. Although PMF is a well-established receptor model for identifying heavy metal sources, its performance is influenced by uncertainty estimation, the choice of factor count, and model configuration (58). Consequently, the source apportionment results presented here should be interpreted as reasonable inferences regarding potential contamination sources rather than definitive source identifications (59, 60).

## 5 Conclusion

In this study, we examined heavy metal exposure in Guizhou snub-nosed monkeys in the FMWNHS. The results obtained confirmed that fecal analysis is a simple, safe, and non-invasive approach for monitoring environmental pollutant exposure and its effects on threatened species. Further, Cu showed relatively high concentrations in the analyzed samples as well as distinct enrichment characteristics across all the sampling sites. However, it showed relatively ecological risks. Conversely, the other heavy metals showed varying concentrations according to site. Natural activities were identified as the primary source of Cu, whereas atmospheric deposition was identified as the primary source of the other heavy metals. Ecological risk assessment indicated moderate to very high potential ecological risk levels, with higher risks at NJD and LJW. Additionally, *RI* analysis identified Hg and Cd as the primary potential ecological risk factors affecting the species. However, all six heavy metals analyzed were within the acceptable exposure range and posed a low potential risk of adverse health effects to Guizhou snub-nosed monkeys.

These findings provide a reliable framework for monitoring contaminant exposure in rare and endangered species and underpin risk assessment and early-warning management of their habitats. Long-term, systematic biomonitoring is recommended in high-risk areas, particularly NJD and LJW, paired with source apportionment to identify and mitigate major pollutant input pathways. Integrating fecal monitoring into routine conservation programs would enable ongoing population health assessment and adaptive conservation strategies, thereby improving long-term survival prospects for these endangered species.

## Data Availability

The raw data supporting the conclusions of this article will be made available by the authors, without undue reservation.
